# Cohesive and anisotropic vascular endothelial cell motility driving angiogenic morphogenesis

**DOI:** 10.1038/s41598-019-45666-2

**Published:** 2019-06-26

**Authors:** Naoko Takubo, Fumitaka Yura, Kazuaki Naemura, Ryo Yoshida, Terumasa Tokunaga, Tetsuji Tokihiro, Hiroki Kurihara

**Affiliations:** 1 Department of Physiological Chemistry and Metabolism, Graduate School of Medicine, The University of Tokyo, 7-3-1, Hongo, Bunkyo-ku, Tokyo 113-0033 Japan; 2 Core Research for Evolutional Science and Technology (CREST), Japan Science and Technology Agency (JST), Chiyoda-ku, Tokyo 102-0076 Japan; 3 Department of Complex and Intelligent Systems, School of Systems Information Science, Future University Hakodate, 116-2 Kamedanakano-cho, Hakodate Hokkaido, 041-8655 Japan; 4 The Institute of Statistical Mathematics, Research Organization of Information and Systems, 10-3 Midori-cho, Tachikawa, Tokyo 190-8562 Japan; 5 Faculty of Computer Science and Systems Engineering, Kyushu Institute of Technology, 680–4 Kawazu, Iizuka Fukuoka, 820-8502 Japan; 6 Interdisciplinary Center of Mathematical Sciences (ICMS), Graduate School of Mathematical Sciences, The University of Tokyo, 3-8-1 Komaba, Meguro-ku, Tokyo 153-8914 Japan; 7 Present Address: Isotope Science Center, The University of Tokyo, 2-11-16, Yayoi, Bunkyo-ku, Tokyo 113-0032 Japan

**Keywords:** Angiogenesis, Collective cell migration

## Abstract

Vascular endothelial cells (ECs) in angiogenesis exhibit inhomogeneous collective migration called “cell mixing”, in which cells change their relative positions by overtaking each other. However, how such complex EC dynamics lead to the formation of highly ordered branching structures remains largely unknown. To uncover hidden laws of integration driving angiogenic morphogenesis, we analyzed EC behaviors in an *in vitro* angiogenic sprouting assay using mouse aortic explants in combination with mathematical modeling. Time-lapse imaging of sprouts extended from EC sheets around tissue explants showed directional cohesive EC movements with frequent U-turns, which often coupled with tip cell overtaking. Imaging of isolated branches deprived of basal cell sheets revealed a requirement of a constant supply of immigrating cells for ECs to branch forward. Anisotropic attractive forces between neighboring cells passing each other were likely to underlie these EC motility patterns, as evidenced by an experimentally validated mathematical model. These results suggest that cohesive movements with anisotropic cell-to-cell interactions characterize the EC motility, which may drive branch elongation depending on a constant cell supply. The present findings provide novel insights into a cell motility-based understanding of angiogenic morphogenesis.

## Introduction

Angiogenesis is a process of new blood vessel formation from pre-existing vessels by sprouting and intussusception^1,2^. It involves morphogenetic collective movement of endothelial cells (ECs), leading to branching networks by sprouting, elongation and bifurcation. In addition to vessel formation during embryogenesis, angiogenesis occurs in response to tissue ischemia or increased oxygen demand during various conditions such as inflammation, wound healing, menstrual cycle and tumor growth. Therefore, elucidation of mechanisms underlying collective cell migration in angiogenesis is essential to expand our knowledge with regard to these (patho-)physiological events.

Collective cell migration during morphogenesis is often driven by the leading cells, which are accompanied by other following cells that uniformly migrate^3^. As for angiogenic morphogenesis, leading the way has been thought to be an EC called a ‘tip cell’, which responds to pro-angiogenic signals represented by vascular endothelial growth factor (VEGF) presents the Notch ligand delta-like ligand 4 (Dll4) to the following stalk cells^4–8^. However, recent development of single cell imaging technique has revealed that cell migration during morphogenesis involves complex behavior, rather than in a uniform manner^9–11^. In the process of angiogenesis, ECs exhibit dynamic and heterogeneous collective movement referred to as “cell mixing”, in which ECs move forwards and backwards, overtaking each other even at the tip position^12,13^. This phenomenon occurs during *in vivo* angiogenesis as evidenced by labeling experiments on mouse retinal angiogenesis^13^.

Corresponding to these findings, it has been extensively investigated how inhomogeneous collective cell migration elaborately forms tissues and organs. With regard to angiogenesis, it has been reported that the balance and dynamics of ongoing Dll4/Notch signaling between cells play an important role in the rearrangement of tip and stalk cells^14^. The regulation of Dll4/Notch signaling by differential VE-cadherin dynamics, glycolysis and protein kinase A (PKA) may be involved in these dynamics^15–17^. In addition, cell migration of ECs depends on a variety of factors such as microenvironment, chemotaxis and cell-to-cell interaction^18^.

Even though new insights in the field of angiogenesis have been put forward, intrinsic properties of individual cell movements in cell mixing have not been fully understood. Previous in silico analyses suggest that the rearrangement can be largely attributable to stochastic movement of cells^19,20^. By contrast, our recent deterministic models fit well some aspects of collective cell migration during angiogenesis^21,22^, indicating the existence of prevailing cellular interaction which remains unclear. This model incorporates distance-dependent attractive and repulsive two-body interactions into a one-dimensional Newtonian equation of motion defined by discrete equations. Giving cell density-dependent conditions for elongation and bifurcation, it successfully reproduces branching morphogenesis with cellular mixing and U-turn behavior. Although this model has succeeded in explaining complex EC behaviors by cell-to-cell interactions based on simple Newtonian dynamics, the assumption of two-body interactions is not quantitatively validated. Furthermore, anisotropic nature of the two-body interactions, which may be caused by cellular polarity, is not considered.

In this study, we aimed at characterizing the nature of EC movement, which gives a driving force to generate branch elongation during angiogenesis. For this purpose, we applied an automated cell tracking system to collect data from an *in vitro* isolated sprout model. This approach revealed different global patterns of EC movement during branch elongation and effects of cell supply from the base of branches. Isolated branches without cell supply showed linear reciprocating movement of ECs. Validation of our mathematical model based on this movement indicates anisotropic nature of cell-to-cell interactions, which better explains EC movement during branch elongation. These results provide novel insights into understanding why ECs can specifically form branching structures through angiogenic processes.

## Results

### Time-lapse live imaging of ECs in branching morphogenesis

**Figure 1:**
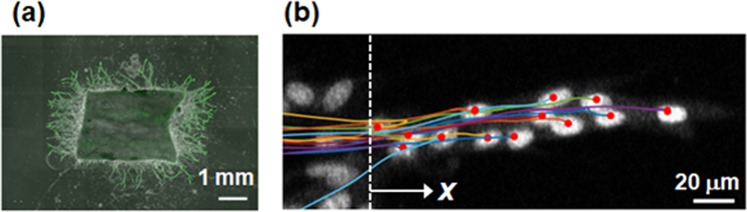
(**a**) Fluorescence (green) and phase-contrast fusion image of branch structures that sprout from a mouse aortic explant at day 7. (**b**) Fluorescence image and tracking of individual EC nuclei in an elongating branch using the tracking system. Dotted line shows base line (*x* = 0). Position and trajectory of each EC are depicted by red point and colored line, respectively.

To investigate vascular EC movement during angiogenic morphogenesis, we examined time-lapse imaging of ECs using the mouse aortic sheet assay, an *in vitro* angiogenic model modified from the aortic ring assay^13,23^ (See Material and Methods). Figure 1(a) shows a representative image of branching structure in the mouse aortic sheet assay at day 7, which mimics angiogenic morphogenesis. Despite a number of surrounding non-EC cells including mural cells, the nuclei of ECs were selectively visualized by live staining with the green fluorescent dye SYTO 16^13^. In this assay, proliferating ECs first formed a cell sheet around the aortic explant. Then, multicellular protrusions sprouted out radially from the aortic sheet to form a complex branching network structure after elongation and bifurcation.

In this study, individual EC movements within a simple branch structure around the distal tip region without lumenization were focused on, although this assay reproduces various important modules of angiogenic processes including bifurcation and lumenization in the proximity of the basal sheet^13,19^. Specifically, ECs were tracked for 36 hours during linear branch elongation after sprouting from the EC sheet in the presence of VEGF, which has been shown to enhance anterograde motility and vessel elongation without changing the frequency of exchanging tip cell position in a dose-dependent manner^13^. Since VEGF was uniformly present in the culture medium, the effect of regional gradients of VEGF concentration on cell movement was considered negligible. Then, individual EC movement in elongating branches were quantitatively analyzed by using the automated cell tracking system based on MATLAB, which we developed. Figure 1(b) shows a representative tracking of individual EC nuclei using this system. The *x*-axis in the figure is set along the direction of branch elongation from a basal sheet (*x* = 0). It is noted that overlapped nuclei were correctly discriminated from each other by using the tracking system. Moreover, individual ECs were also identified in cases when an EC passed another one. Representative fluorescence movie tracking individual EC nuclei in an elongating branch is shown in Supplementary Movie S1.

### Overall dynamics of ECs in branch elongation

Time series data of each EC position were obtained from time-lapse images of 5 different branches and were subjected to analysis. Not only position but also velocity of each cell nucleus was estimated using the developed tracking system. Here, the EC position was defined as that of the centroid of each EC nucleus.

**Figure 2:**
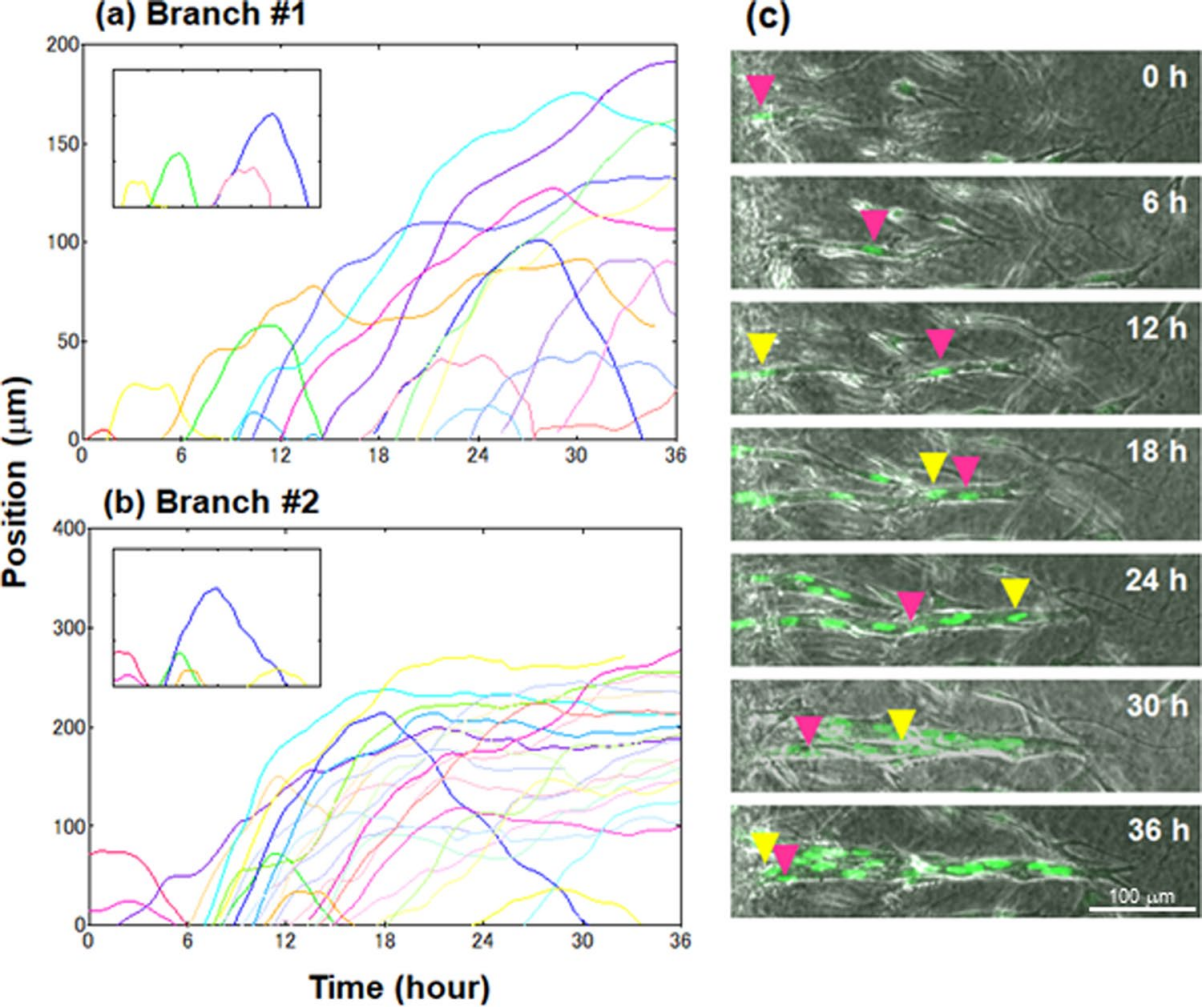
Time evolution of individual EC positions along each elongating branch (*x*-axis) #1 (**a**) and #2 (**b**). Each line with different color represents the trajectory of individual EC. Replotted data with regard to U-turn cells are shown in the insets. (**c**) Time-lapse images of ECs including a U-turn cell in elongating branch observed for 36 hours. Each image is a superimposition of phase-contrast and fluorescence microscopy (green) ones. Red and yellow arrows indicate tip and following cells, respectively.

Figure 2(a,b) show time evolution of EC positions along each elongating branch #1 and #2. As observed previously^13,19^, inhomogeneous cell movements were evident with frequent overtaking and cell mixing, resulting in replacement of tip cells several times within 36 hours in both records. We then start the analysis by characterizing the patterns of cell behaviors during the branch elongation process.

During the observation, many cells were “ongoing”, that is, unidirectionally moving forward except for small fluctuation less than 20 μm, which is comparable to the long axis of a nucleus. Remarkably, some ECs moved forward and then turned back to the base, which we hereafter refer to as “U-turn” cells. The numbers of U-turn cells in five branches are listed in Table 1. Ongoing cells might have a potential to become U-turn cells in longer observation time (>36 h). Fluorescence movie and tracking of the nucleus of a U-turn cell in an elongating branch using the tracking system is shown in Supplementary Movie S2.

**Table 1:** Number of total/U-turn/ongoing cells in five branches and forward/backward velocity of each U-turn cell calculated by fitting line to the insets in Fig. 2(a,b).

		Branch				
		#1	#2	#3	#4	#5
Number of total cells		14	25	11	23	21
Number of U-turn cells		4	6	3	5	6
Number of ongoing cells		9	16	7	18	13
**Velocity of U-turn cell**						
#1	Forward	23.7	N/A	N/A	N/A	11.2
	Backward	12.4	24.1	6.8	15.6	8.4
#2	Forward	15.2	N/A	13.9	8.0	23.6
	Backward	25.3	9.8	N/A	N/A	18.1
#3	Forward	11.5	23.5	1.8	13.8	16.1
	Backward	17.2	23.6	7.4	N/A	N/A
#4	Forward	9.2	29.2	N/A	17.7	11.1
	Backward	13.1	16.9	N/A	18.9	18.8
#5	Forward	N/A	22.0	N/A	23.9	18.0
	Backward	N/A	17.8	N/A	N/A	N/A
#6	Forward	N/A	8.3	N/A	N/A	14.4
	Backward	N/A	7.0	N/A	N/A	N/A
Mean ± SD (μm/hour)	Forward	14.9 ± 6.4	20.7 ± 8.9	7.8 ± 8.6	15.8 ± 6.7	15.8 ± 4.7
	Backward	17.0 ± 5.9	16.5 ± 7.0	7.1 ± 0.4	17.2 ± 2.3	15.1 ± 5.8

In this table, “U-turn cells” are specifically defined as those that turn back more than half way to the base during the measurement period. “Ongoing” is defined as unidirectionally moving forward. Note that small movement a cell less than 20 μm, which is comparable to the long axis of a nucleus, is regarded as fluctuation. Note that dead cells were excluded from number of total cells.

As shown in Fig. 2(a,b) with replots in the insets, forward and backward velocities are generally constant among U-turn cells. Time evolution of EC positions in branch #3 to #5 also gave similar results (Supplementary Fig. S1). These were estimated by fitting line to the insets, as listed in Table 1. It is also notable that forward and backward velocities of each U-turn cell are similar to each other.

Figure 2(c) shows time-lapse images of ECs including U-turn cells in an elongating branch observed for 36 hours. Each image is a superimposition of phase-contrast and fluorescence microscopy ones. As shown in the figure, typical behavior of a U-turn cell is as follows: (1) a tip cell indicated by a red arrow is moving forward with a constant velocity (0–12 h), (2) slowing down (12–18 h), (3) moving backward to the base with a constant velocity right after the following cell indicated by a yellow arrow comes close to the tip cell (18–36 h). It is thus suggested that the U-turn phenomenon of a tip cell is triggered by some interaction with the following cell. In turn, the following cell likely act as a tip cell to elongate the branch with high forward velocity. The turning points of observed U-turn cells were mainly (~70%) located either at the tip region including the second or third position, or near bifurcation sites as well.

### Branch elongation depends on regional increases in immigrating cell numbers

**Figure 3:**
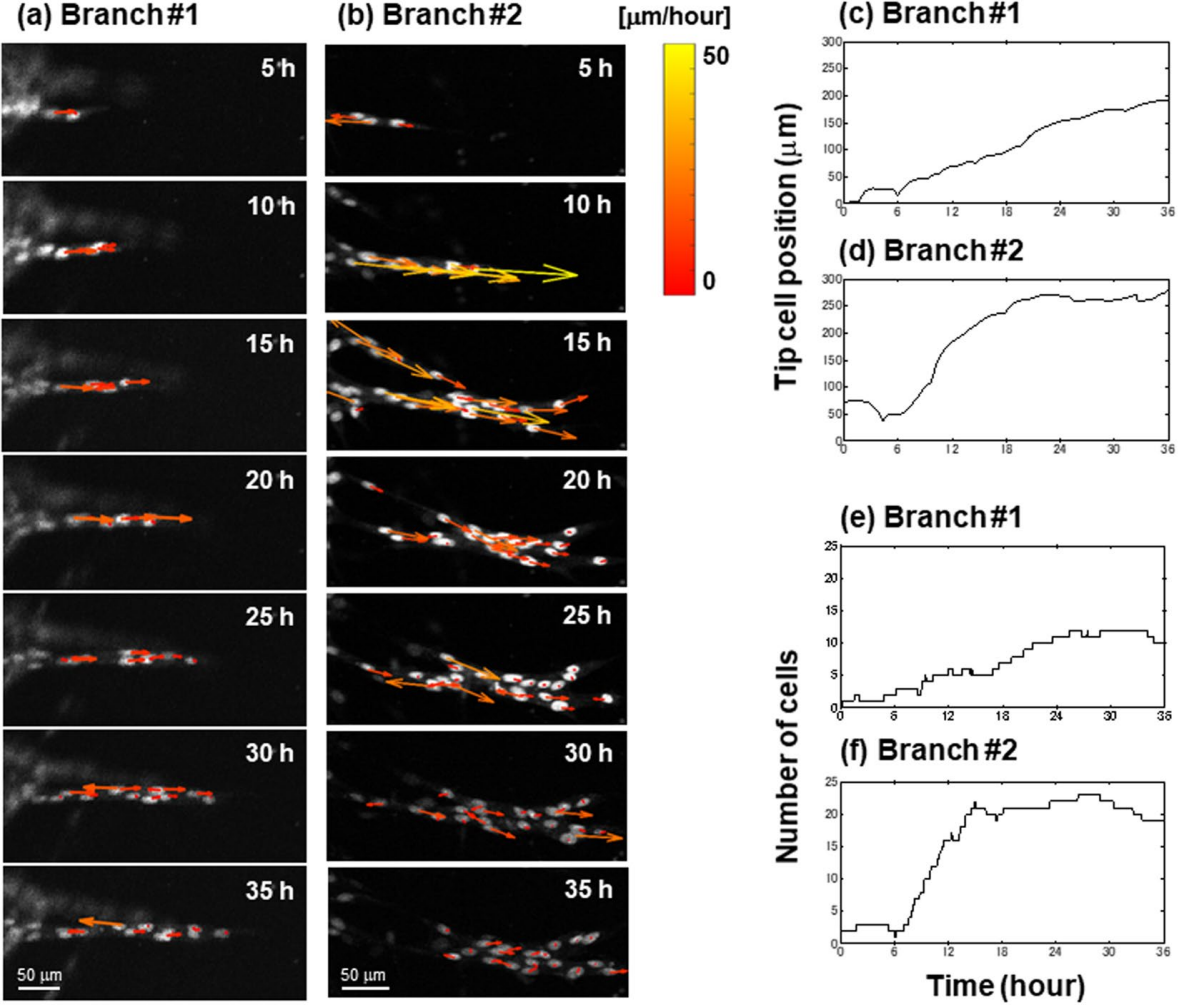
Time-lapse images of cell nuclei in branches #1 (**a**) and #2 (**b**) obtained by fluorescence microscopy. An arrow on a cell nucleus shows a velocity vector. Time evolution of tip cell position in branches #1 (**c**) and #2 (**d**). Time evolution of number of cells in branches #1 (**e**) and #2 (**f**).

We then focus on collective cell movement in branch elongation. Figures 3(a,b) show time-lapse images of cell nuclei in branches #1 and #2 obtained by fluorescence microscopy. An arrow on each cell nucleus shows a velocity vector. Figures 3(c,d) show positions of a tip cell in branches #1 and #2, respectively. Figures 3(e,f) show the number of cells in branches #1 and #2.

With regard to branch #1, it elongates with a constant velocity of about 6 μm/hour, as shown in Fig. 3(c). Most ECs in branch #1 showed constant velocity around 5–10 μm/hour throughout observation (Figs 2(a) and 3(a)). On the other hand, branch #2 elongated at around 6–18 hours with velocity up to 50 μm/hour at around 10 hours (Figs 2(b) and 3(b,d)). However, branch elongation was stagnant after 18 hours after the steep rise of elongation. During this stagnant period, most of the ECs in branch #2 moved only slowly. These observations suggest that branch elongation may depend on collective cell movement representing relatively uniform flow of ECs in a branch.

Here, we focus on the number of cell inflow from the basal sheet into the forming branch. Since cell division was seldom observed within the branch, increase of the number of cells was supposed to be due to the cell migratory inflow from the sheet. According to Fig. 3(e), the number of cell inflow in branch #1 was almost constant. On the other hand, experimental data with regard to branch #2 showed a large number of cell inflow when it remarkably elongated between 6 and 18 hours (Fig. 3(f)). Then, the position of tip cell, namely, branch elongation ceased after 18 hours, coincided with saturation of the number of cells, or negligible cell inflow.

It is also notable that the branch #2 showed inhomogeneous distribution of ECs after 20 hours, where most of the cells were in the vicinity of the tip, forming bifurcation into two or more branches, as shown in Fig. 3(b). By contrast, the branch #1 showed homogeneous distribution of ECs throughout observation period, forming a single branch with narrow structure about two cells in width, as shown in Fig. 3(a). These experimental results suggest that cell inflow from the aortic sheet may be a determinant of branch elongation and bifurcation as well.

### ECs exhibit cohesive linear to-and-fro movements in an isolated branch

**Figure 4:**
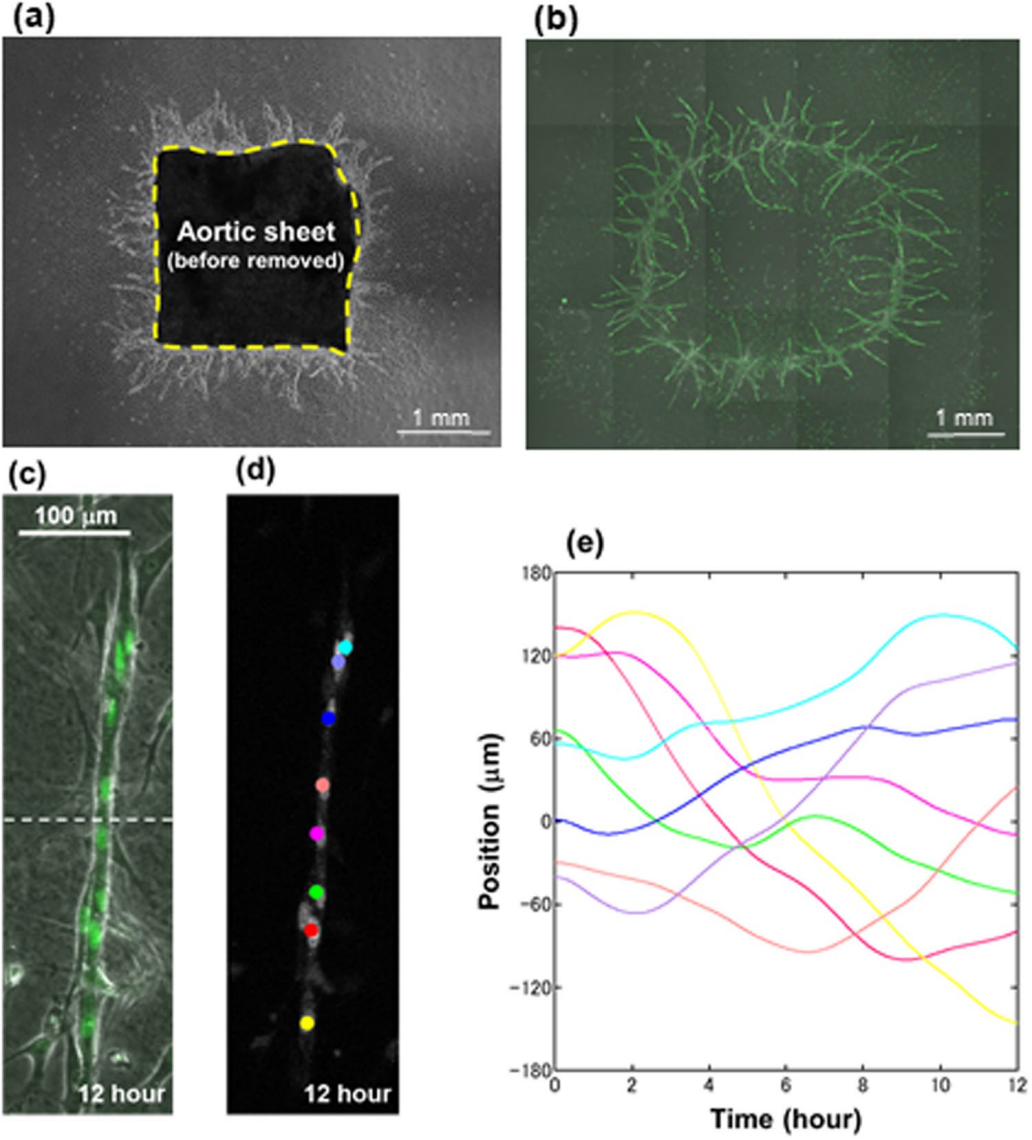
Aortic sheet removal assay. (**a**) Phase-contrast image of aortic sheet from which branches are radially sprouting. (**b**) Fluorescence (green) and phase-contrast fusion image of branch structures three days after removal of aortic sheet removal, as shown in Fig. 4(a). Note that new branches are sprouting within the cutting line toward opposite direction from the former sprouting before removal of aortic sheet. (**c**) Fluorescence and phase-contrast fusion image of isolated branch at 12 hours. Dotted line shows original boundary between an aortic sheet and new branches. (**d**) Tracking of individual EC nuclei on fluorescence image of isolated branch at 12 hours. (**e**) Time evolution of cell positions in the isolated branch for 12 hours. Each line with different color represents trajectory of individual EC.

To delineate intrinsic properties of individual cell movement in a branch, it would be necessary to eliminate the effect of cell inflow that may affect both branch elongation and collective cell movement as described above. For this purpose, we established an aortic sheet removal assay, in which isolated branches are cultured in the absence of cell inflow by removing the proximal sheet on the four or fifth day of the aortic sheet assay, when new branches elongated up to 1 mm (Fig. 4(a)). The aortic sheet was then replaced with added collagen gel in a culture dish, in which branches sprouting outward from the sheet remained intact. After removal of the aortic sheet, most branches grew inward from the root position, resulting in a characteristic mirror image of the preexisting branch (Supplementary Fig. S3(a–d), Movies S3(a–c)). It is thus noted that a mound of ECs moved beyond the root position after excision of the aortic sheet.

Since cell proliferation mainly occurred within the aortic sheet, increases of ECs due to proliferation were negligible for the new branches after excision of the aortic sheet. Therefore, individual cell movement could be observed in the isolated branch model without cell inflow that might influence collective cell movement. Fluorescence movies of individual EC nuclei in each isolated branch are shown in Supplementary Movies S4(a–e).

Figure 4(c) shows a representative image of an isolated branch after excision of the aortic sheet. The isolated branch consisted of eight cells in this case. The dotted line shows an original root position between an aortic sheet and sprouting branches. Figure 4(d) shows tracking of individual EC nuclei superimposed on a fluorescence image of isolated branch at 12 hours. According to Fig. 4(b–d) and Supplementary Fig. S3(b–f), the isolated branch showed relatively homogeneous distribution of ECs, forming a thin branch structure with one or two cells in width.

Figure 4(e) shows time evolution of cell positions in the isolated branch for 12 hours. The time of onset is defined by 6 hours after excision of the aortic sheet. The eight cells moved along the direction of branch elongation. The number of cells was constant during the observation. It was observed that five cells turn at either end of the branch in the observation time (12 h) of the experiment. The five cells can be regarded as U-turn cells. Supplementary Figure S3(e) shows tracking of individual EC nuclei on fluorescence image of isolated branch for 12 hours. It is remarkable that half the ECs in the isolated branch were moving forward and the other half backward. In addition, the ECs were U-turning at one end in sequential. Accordingly, Fig. 4(e) and Supplementary Figure S3(e) clearly show linear reciprocating movement of ECs in angiogenesis. These results indicate that, in the absence of cell supply, ECs keep contact each other to continue linear to-and-fro movements with equivalent velocities toward both directions.

### A mathematical model considering the anisotropic nature of two-body interactions well describes collective EC behaviors during angiogenesis

To assess the biological validity of our deterministic mathematical model for cellular dynamics in angiogenetic morphogenesis^21^ and elaborate it, we analyzed the present data in terms of the fitness to the model. We first extended this model to a two-dimensional one as follows,

1 $${{\boldsymbol{v}}}_{n}^{t+1}-{{\boldsymbol{v}}}_{n}^{t}=-\,\gamma {{\boldsymbol{v}}}_{n}^{t}+\sum _{k\ne n}F(\Vert {{\boldsymbol{z}}}_{n}^{t}-{{\boldsymbol{z}}}_{k}^{t}\Vert )\frac{{{\boldsymbol{z}}}_{n}^{t}-{{\boldsymbol{z}}}_{k}^{t}}{\Vert {{\boldsymbol{z}}}_{n}^{t}-{{\boldsymbol{z}}}_{k}^{t}\Vert }$$

where ${{\boldsymbol{z}}}_{n}^{t}$ and ${{\boldsymbol{v}}}_{n}^{t}={{\boldsymbol{z}}}_{n}^{t+1}-{{\boldsymbol{z}}}_{n}^{t}$ are position and velocity of *n*th EC at time *t*. The force term is isotropic, which depends only on the relative positions ${{\boldsymbol{z}}}_{n}^{t}-{{\boldsymbol{z}}}_{k}^{t}$ between two ECs. The present experimental data suggested that little inflow of cells give rise to stagnant movements. For this effect, we introduced a relaxation term $\gamma {{\boldsymbol{v}}}_{n}^{t}$ in the equation.

**Figure 5:**
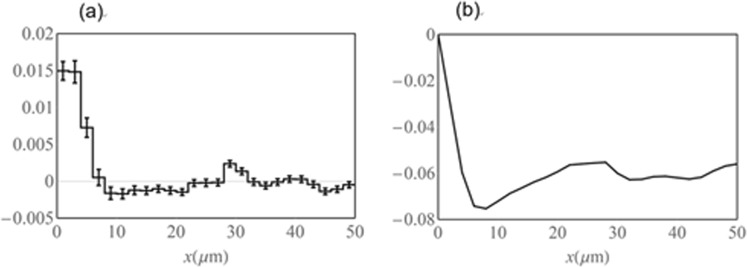
(**a**) Estimated force *F*(x) of branch #2 with *R*_*d*_ = 50, *N* = 25. (**b**)$\,-{\int }_{0}^{x}F(\mu )d\mu$, potential of (**a**).

**Figure 6:**
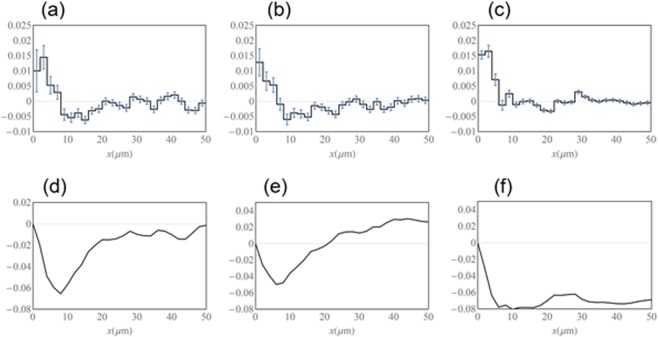
Estimated forces (**a**) *F*_1_(*x*), (**b**) *F*_2_(*x*), and (**c**) *F*_3_(*x*). These functions were obtained in accordance with experimental data of branch #2. (*R*_*d*_ = 50, *N* = 25). Potentials of each forces, (**d**) $-{\int }_{0}^{x}{F}_{1}(\mu )d\mu$, (**e**) $-{\int }_{0}^{x}{F}_{2}(\mu )d\mu$, (**f**) $-{\int }_{0}^{x}{F}_{3}(\mu )d\mu$, respectively.

When anisotropic nature of the cellular interactions was not considered, time-series data of multicellular movement from two independent experiments gave similar estimation of parameters, fitting to the assumption of distance-dependent interactions; with repulsive force in ~8 μm and attractive force in 8~30 μm (Fig. 5). Since each EC has its own volume, proximity of two cells may produce the repulsive force as a result of excluded volume effects. On the other hand, the attractive force may reflect contact-dependent acceleration. Accordingly, we considered anisotropy of cellular interactions. The presence of U-turn cells moving backward produces different pattern of directionality as follows: (i) two ECs move into the opposite direction apart from each other; (ii) two ECs move into the opposite direction approaching each other; (iii) two ECs move in the same direction. Based on these classifications, we extended Eq. (1) to Supplementary Equation (S4). Three force functions *F*_1_, *F*_2_, and *F*_3_, which correspond to the three patterns (i), (ii), and (iii), respectively, were obtained from analysis on experimental data of branch #2 (Fig. 6). In *F*_1_ and *F*_2_, both repulsive (~8 μm) and attractive (8~30 μm) components were evident. By contrast, *F*_3_ contained only a repulsive component in ~8 μm, with no evident attractive component. Figure 6(d–f) are the integrals of Fig. 6(a–c) respectively, that is, potential. While Fig. 6(d,e) clearly indicate the positive slope (8~30 μm), Fig. 6(f) shows relatively flat in the region 6~50 μm. In Figure S6, the differences of three functions *F*_1_, *F*_2_ and *F*_3_ are plotted with standard deviation of the estimated differences. Since we assume multiple linear regression with Gaussian noise (see also Supplementary Information), the estimated parameters and their difference are also in accordance with normal distribution. Figures S6(b,c) show the differences, *F*_1_ –*F*_3_ and *F*_2_ –*F*_3,_ are negative in almost all regions, where the error bar is 68% confidence interval independently. Hence, it is suggested that an attractive interaction occurs between a pair of a U-turn cell and a cell moving forward, resulting in accelerated cell movement when passing each other. Conversely for lateral cells, attractive force is not much induced. In Supplementary Information, we show the estimations of anisotropic forces for other than the branch #2 (Supplementary Figs S7–S10). The positive slopes are prominent for *F*_1_ and *F*_2_ compared to *F*_3_ in the region 8–20 μm in most cases.

To test whether estimated functions reflect the EC dynamics in branching morphogenesis, we simulated the time evolution of our mathematical models based on estimated parameters. In Figure S11, a simulated result by Supplementary Equation (S4) with the estimated functions is shown. As a boundary condition perpendicular to elongation, we limit the width of *y*-direction in which the cells exist to 20 μm, which satisfy the experimental settings in the process of elongating morphogenesis bounded by surrounding collagen gel. The estimated functions reproduced collective cell movement with mixing and U-turn within a steady span of trajectory, which was in good agreement with the experimental results represented in Fig. 4(e).

## Discussion

In the present study, we characterized EC movement during *in vitro* branch elongation mimicking angiogenesis and identified different global patterns of EC movement including backward U-turn movement. According to the experimental results, there are four features in the U-turn movement. First, a U-turn cell typically turns at either the tip or bifurcation regions of a branch. Namely, a U-turn cell directionally moves forward to the turning point and then moves backward to the base in a sprout. Secondly, backward velocity of a U-turn cell is comparable with forward velocity. Moreover, experimental results using an isolated branch model suggest that U-turn movement is independent of chemotaxis. Even in the case of angiogenesis, forward movement of an EC is thus essentially the same as backward movement. Thirdly, a U-turn cell slows down near the tip or bifurcation regions. Fourthly, a U-turn cell starts to move backward concomitantly with the approach of a forward-moving following cell.

This U-turn movement, together with the linearly aligned movement, manifested as linear reciprocating movement of ECs in an isolated branch composed of an invariant number of cells. This observation suggests that the linearly aligned movement and U-turn constitute the intrinsic nature of ECs with angiogenic properties. This experiment also supports the idea that branch elongation accomplished by ever interchanging tip cells depends on cell supply from the base of branches, which validates the design of our mathematical model of angiogenesis based on two-body interactions^21^. Because this model assumes that the sum of intercellular action-reaction forces drives the movement of each cell, the centroid of the whole cell system should be unmoving unless cells are supplied from the outside of the system. Thus, lack of further branch elongation in the absence of cell supply in the isolated branch is consistent with the assumption of our mathematical model in this respect.

Furthermore, data analysis indicated distance-dependent intercellular forces; a repulsive force in ~8 μm and attractive force in 8~20 μm, which well corresponded to our mathematical model^21^. When anisotropy of cellular interactions was considered, an attractive interaction was observed between cells moving in opposite directions, that is, between a pair of a U-turn cell and a cell moving forward. Incorporation of this condition into the mathematical model resulted in the simulation of EC behaviors in an isolated branch better than the former isotropic model. Therefore, the present experimental results apparently validate the assumption of the intercellular action-reaction forces and refine the model to better simulate the EC behaviors.

The result that two components of the EC behavior in forming branch structures, directional linear movement and U-turn, could be well simulated by our model with a modification to include anisotropy of cellular interactions may indicate that these characteristic movements may be mediated by contact-dependent molecules whose activities are differently localized along the leading (anterior)-trailing (posterior) axis. In migrating ECs, Cdc42-dependent sensing of the motile stimuli by filopodia and Rac1-dependent formation of lamellipodia take place in the leading edge, while Rho-dependent stress fiber contraction and rear release occur in the trailing edge^18^. Distribution of cell surface molecules are also affected by these differences. Thus, anisotropic differences in intracellular forces may be due to interactions between different cell surface structures and molecules, determining the mode of cellular behaviors.

In general, migratory cells exhibit contact inhibition of locomotion, in which Rac1-dependent lamellipodial protrusions are inhibited upon cell-cell contact, resulting in cell repolarization and new protrusion formation away from the contact to separate cells from each other^24,25^. By contrast, ECs tend to keep contact with each other, aligning along the common direction of branch elongation. This cohesive nature and anisotropic interactive forces may define the characteristics of EC behaviors, which may drive angiogenic morphogenesis depending upon the constant supply of immigrating cells.

## Methods

### Aortic sheet assay

#### Animals

C57/B6Ncr mice were purchased from Japan SLC and used at 9–15 weeks of age in all assay. Animal experiments were reviewed and approved by The University of Tokyo Animal Care and Use Committee and were performed in accordance with the institutional guidelines.

### Aortic sheet assay

We modified the previously described aortic ring assay^13,23^ to observe EC behaviors during sprouting and branching morphogenesis mimicking angiogenesis. Instead of ring-shaped aortic sections, rectangle-shaped aortic explants (~3 × 4 mm) excised from the mouse thoracic aortae were embedded in type I collagen gel (Nitta Gelatin) with the inner lumen side down on glass-bottom culture dishes (Matsunami Glass) and were cultured in medium-199 containing 5% FCS, 10 μg/ml streptomycin, 100 units/ml penicillin and 50 ng/ml human recombinant VEGF (R&D). Medium replacement was performed every other day.

### Aortic sheet removal assay

The aortic sheet removal assay was developed based on the aortic sheet assay. At the four or fifth day of the aortic sheet assay, after removing the culture medium, the aortic explant and the EC sheet existing behind the aortic explant were excised with a scalpel. Collagen gel and culture medium were then added.

### Time-lapse live single cell imaging

Time-lapse imaging was started 3 days or 4 days after the aortic sheet culture. EC nuclei were selectively labeled with fluorescent probe SYTO-16 (50 nM, Invitrogen). Time-lapse fluorescence and phase-contrast images ware obtained every three or five minutes over 36 hours, using a confocal laser scanning microscope (FV-10i Olympus) with 10 × 0.4 NA air objective lens.

### Cell tracking

To quantitatively investigate the dynamic behaviors of cells over time, it was necessary to track many cells moving across the time-lapse 2D image sequences. First we performed an automated cell tracking. In each frame of the time-lapse video, all imaged EC cells were segmented from the background. After that, every segmented cell in any given frame was associated with one of the neighboring cells in the next frame. This task was nontrivial because of several reasons: (i) EC nuclei had quite similar ellipsoidal appearance, and hence it was difficult to use morphological features in the association step; (ii) cells turned over suddenly between forwards and backwards; (iii) ECs overtook or passed each other on a narrow lane of blood vessels; (iv) cell division and birth/death were often observed. To address these issues, we implemented an in-house cell tracking algorithm as detailed in Supplementary Information. Using this, we calculated the trajectories of total 139 cells on arbitrary chosen six lanes of blood vessels in five independent measurements. Finally, tracking failures were detected and corrected by manual trajectory inspection on the image analysis platform using ImageJ^26^.

## Supplementary information

Supplementary Information

Movie S1

Movie S2

Movie S3(a)

Movie S3(b)

Movie S3(c)

Movie S4(a)

Movie S4(b)

Movie S4(c)

Movie S4(d)

Movie S4(e)
