# Supplementary material for: Cohesive and anisotropic vascular endothelial cell motility driving angiogenic morphogenesis
Source: Sci Rep. 2019 Jun 26;9:9304. doi: 10.1038/s41598-019-45666-2 (PMC6594931; doi:10.1038/s41598-019-45666-2)
Supplement: Supplementary file 1 — Supplementary Information [file 41598_2019_45666_MOESM1_ESM.docx]

**Supplementary Information**

**Cohesive and anisotropic vascular endothelial cell motility driving angiogenic morphogenesis**

Naoko Takubo^1,2^, Fumitaka Yura^3^, Kazuaki Naemura^1^, Ryo Yoshida^4^, Terumasa Tokunaga^5^, Tetsuji Tokihiro^2, 6^, and Hiroki Kurihara^1,2^

1) Department of Physiological Chemistry and Metabolism, Graduate School of Medicine, The University of Tokyo, 7-3-1, Hongo, Bunkyo-ku, Tokyo, 113-0033, Japan.

2) Core Research for Evolutional Science and Technology (CREST), Japan Science and Technology Agency (JST), Chiyoda-ku, Tokyo, 102-0076, Japan.

3) Department of Complex and Intelligent Systems, School of Systems Information Science, Future University Hakodate, 116-2 Kamedanakano-cho, Hakodate, Hokkaido, 041-8655, Japan.

4) The Institute of Statistical Mathematics, Research Organization of Information and Systems, 10-3 Midori-cho, Tachikawa, Tokyo, 190-8562, Japan.

5) Faculty of Computer Science and Systems Engineering, Kyushu Institute of Technology, 680–4 Kawazu, Iizuka, Fukuoka 820-8502, Japan.

6) Interdisciplinary Center of Mathematical Sciences (ICMS), Graduate School of Mathematical Sciences, The University of Tokyo, 3-8-1 Komaba, Meguro-ku, Tokyo, 153-8914, Japan.


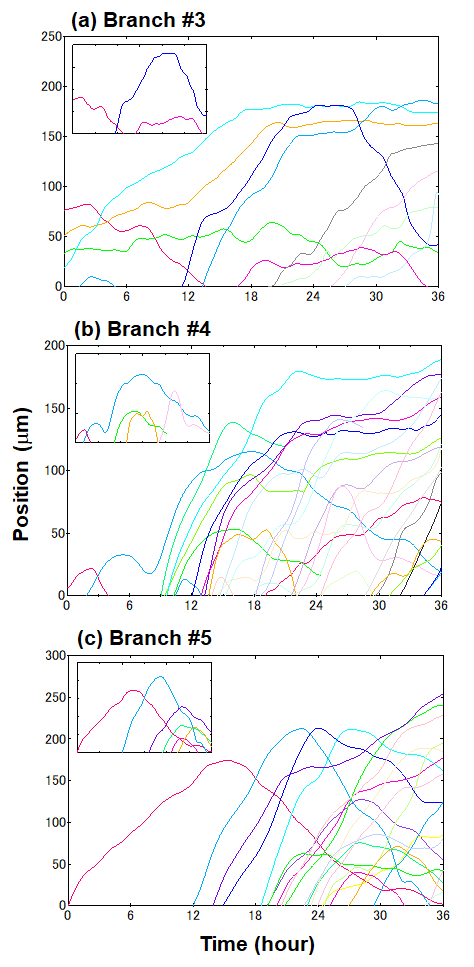


**Figure S1.** Time evolutions of individual EC positions along each branch elongation (*x*-axis) in new branches #3 (a), #4 (b), and #5 (c). Each line with different color represents trajectory of individual EC. Replotted data with regard to U-turn cells are shown in the insets


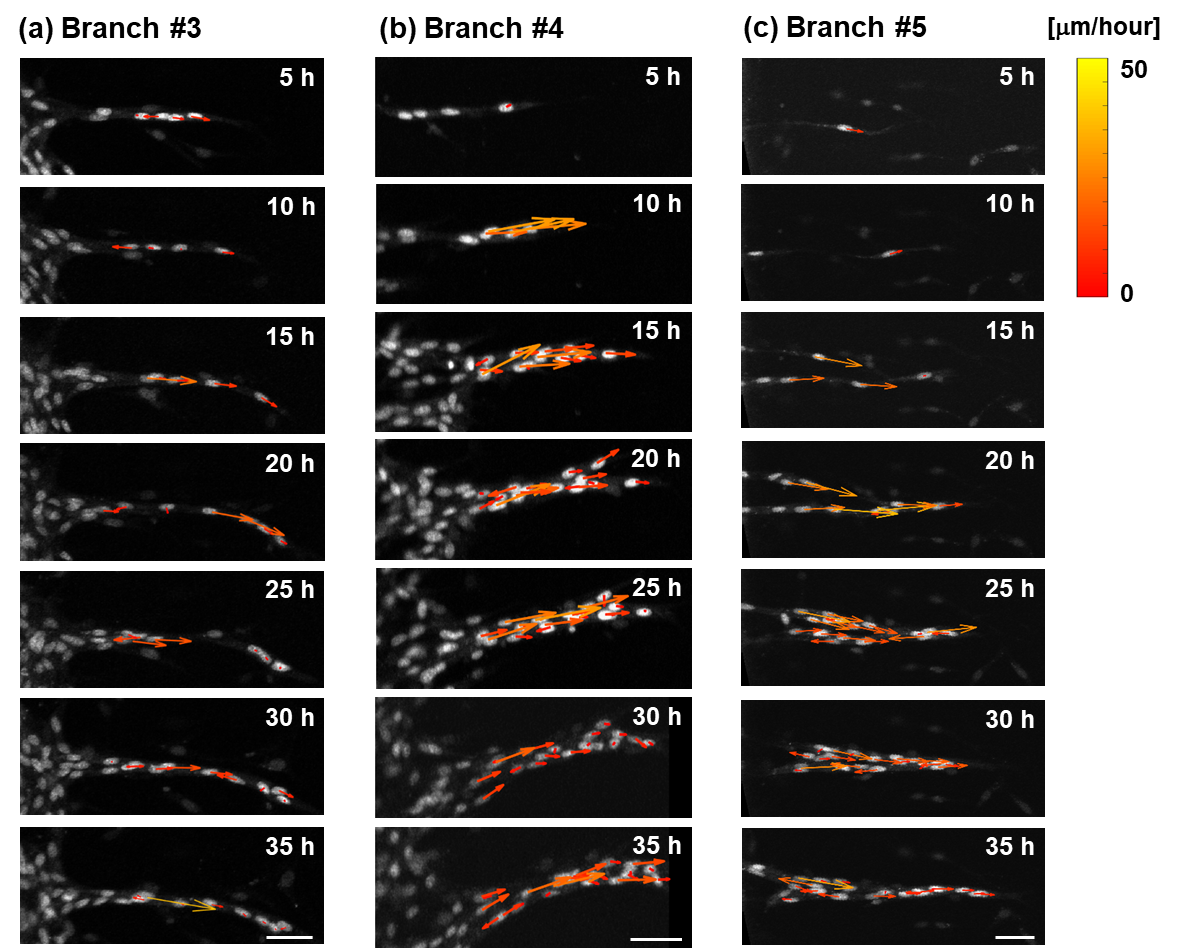

**Figure S2.** Time-lapse images of cell nuclei in branches #3 (a), #4 (b), and #5 (c) obtained by fluorescence microscopy. An arrow on a cell nucleus shows a velocity vector. Scale bars, 50 μm. Time evolution of tip cell position in branches #3 (d), #4 (e), and #5 (f). Time evolution of number of cells in branches #3 (g), #4 (h), and #5 (i).


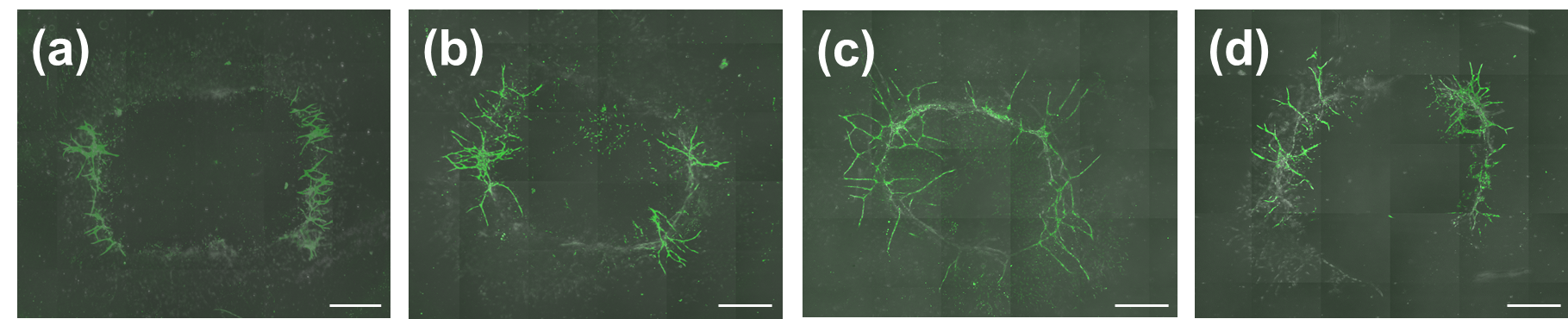


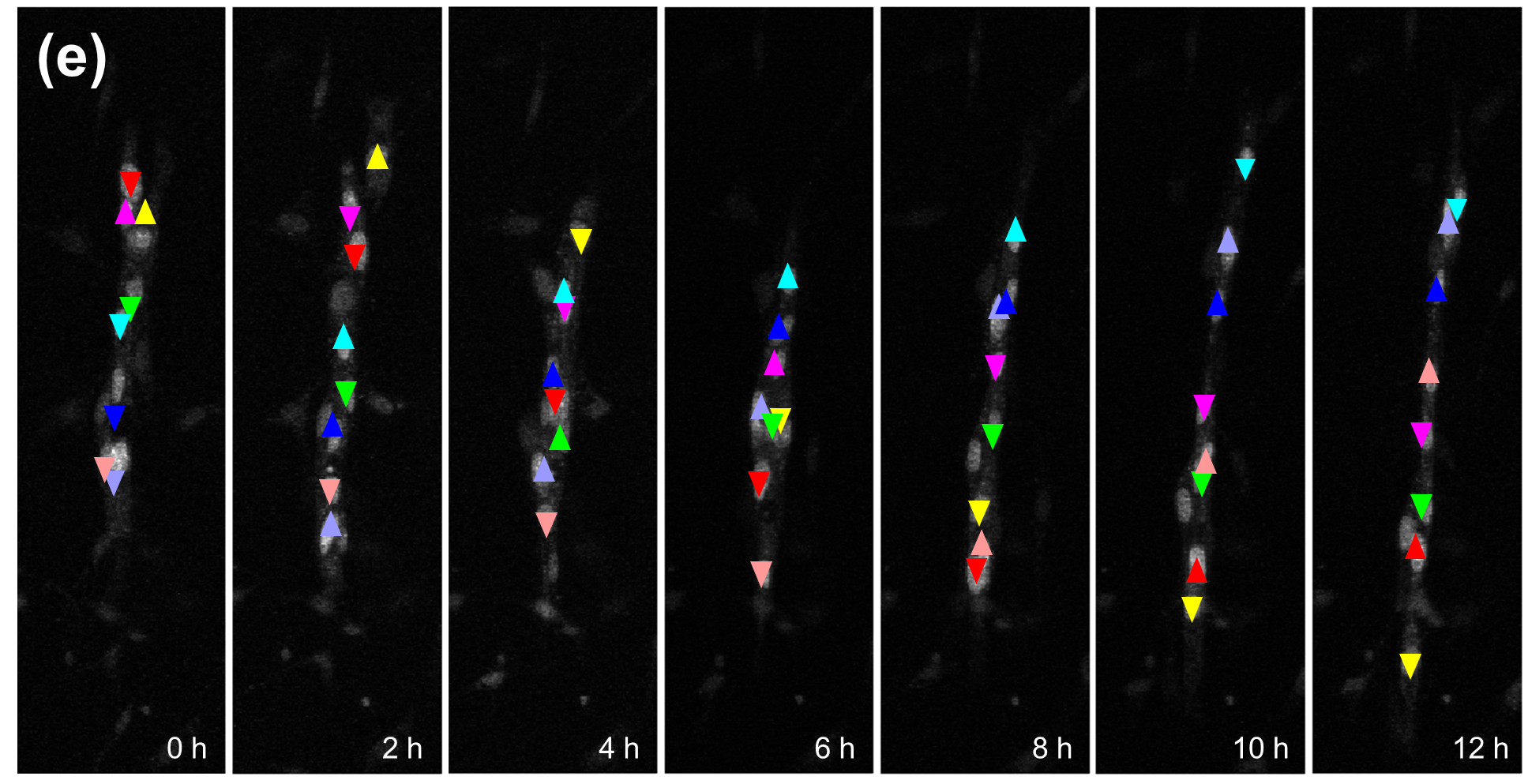


**Figure S3.** (a)-(d) Other examples of branch sprouting in the cases of aortic sheet removal assay. Scale bars, 1 mm. (e) Tracking of individual EC nuclei on fluorescence image of isolated branch for 12 hours. The arrow head represents direction of movement for each ECs at the observation time.

**Segmentation and cell tracking methods.**

First, imaged EC nuclei in each frame were segmented out by performing Algorithm 1. The procedure consists of noise-processing and conventional watershed segmentation [1], followed by the grouping of over-segmented objects in the post-processing step. According to Algorithm 2, the segmented ROIs (region of interest) neighboring between two consecutive time points were linked successively for the determination of cell identities. One difficulty arose from the fact that two or more cells could appear as an overlaid single object when they overtook or passed each others within a narrow lane, as illustrated in Figure S4. In order to reduce identification errors due to such object fusion, the point-cloud matching was performed on the edges of objects rather than their centroids. Then, we conducted one-step ahead prediction of the object centers in the next frame based on the mean shift of the assigned edges.


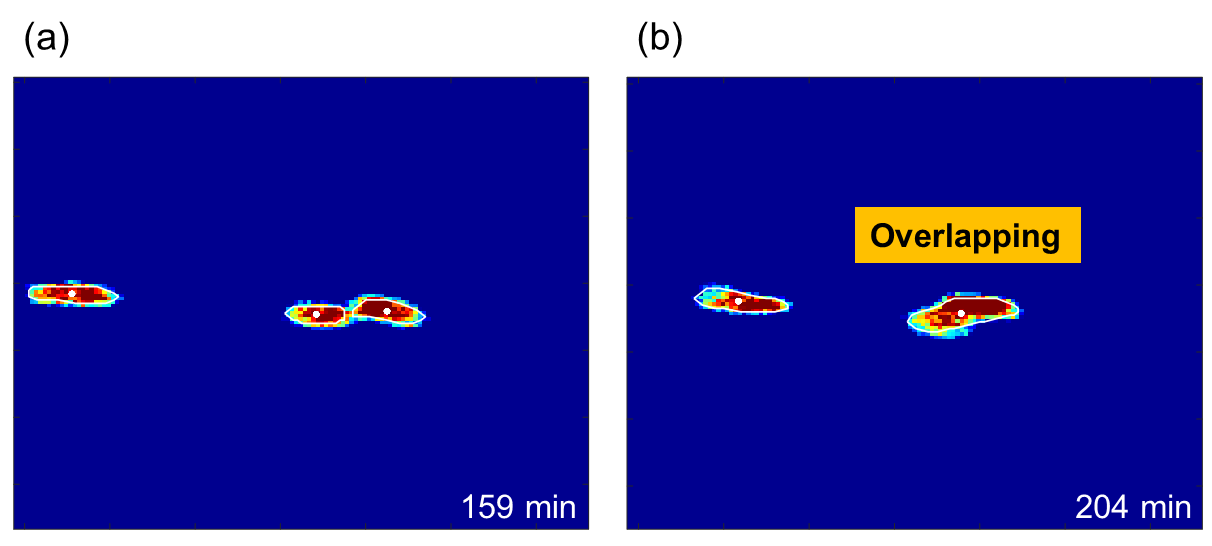


**Figure S4.** Illustrative example of object fusion to be prevented in the object tracking. When a cell overtook another, the two cells were merged into an overlaid single object.

**Algorithm 1: Segmentation** (MATLAB functions are represented in the Consolas font)

**Input:**

2D gray-scale image for a given frame $t$

Threshold values $\varepsilon_{A}$ (= 20), $\varepsilon_{v}$ (= 30), $\varepsilon_{\theta}$ (= 60)

**Output:**

Center positions of ROIs $P_{t}=\left\{ p_{it}|i=1,\cdots,N_{t} \right\}$

Edges of ROIs $E_{t}=\left\{ e_{it}|i=1,\cdots,N_{t} \right\}$ ($e_{it}$ is a set the positions cosisting of the $i$th edge)

**# Noise filtering:**

Apply to the input image the Gaussian blur with the 3-by-3 filter matrix and the standard deviation set to 10 pixel (fspecial('gaussian’)), and then perform a simple mean filter with the 5-by-5 filter matrix (imfilter).

**# Segmentation:**

The watershed algorithm is applied to the filtered image (watershed), and a label is assigned to each segmented region (bwlabel), providing a set of ROIs.

**# ROI center:**

For each ROI $i$, calculate the center position $p_{i}$ (regionprops(ROI, ‘centroid’)).

**# ROI volume:**

Calculate the area size $A_{i}$ of each ROI $i$after applying the convex hull processing (convexhull).

**# Grouping of over-segmented objects:**

**FOR** each pair $\left( i,j \right)$ of ROIs with their centroids positioning within the distance 15 px **DO**

Calculate the area size $A_{ij}$ of the convex hull surrounding the two ROIs.

Calculate the angle $\theta$ between the major axis $v_{ij}$ of the convex hull and　　 $s_{ij}=p_{i}-p_{j}$.

**IF** $A_{ij}-\left( A_{i}+A_{j} \right)\leq\varepsilon_{A}$ and ${\theta\leq\varepsilon}_{\theta}$ **THEN**

The two ROIs are reduced to one by the given convex hull.

**IF** $\left\| v_{ij} \right\|\leq\varepsilon_{v}$, divide the merged ROI into two at the centroid.

**ENDIF**

**ENDFOR**

Remove all ROIs with the area size less than 10.

**Algorithm 2: Cell tracking**

**Input:**

Series of centers of ROIs${\{p}_{it}|i=1,\cdots,N_{t} , t=0,1, \cdots,T\}$

Series of edges of ROIs ${\{e}_{it}|i=1,\cdots,N_{t} , t=0,1, \cdots,T\}$

Radius $r$ (= 25 px) to define a neighboring region

**Output:**

Set $L$ of links between ROIs in two adjacent frames; $\left( i,t-1 \right)\sim\left( j, t \right)$ indicates that ROI $i$ at frame $t-1$ is associated with $j$at $t$.

**# Initialization:**

Indices of active trackers for the initial frame $A_{0}=\left\{ 1,\cdots,N_{0} \right\}$

One-step ahead prediction of ROI centers $\hat{p}_{i1}=p_{i0} \left( i=1,\cdots, N_{0} \right)$

**FOR** $t\in\{1,\cdots,T\}$ **DO**

**FOR** $a\in A_{t-1}$ **DO**

**# Selection of neighbors:**

From $j\in\left\{ 1, \cdots, N_{t} \right\}$ at the current frame $t$, obtain a neighbor set $M_{a}$ of ROI $a$ at $t-1$whose centers are positioned within the radius $r$ from $\hat{p}_{a,t}$.

**# Association:**

**IF** $M_{a}$ is not empty **THEN**

Perform the point cloud matching between edge positions in $\left\{ e_{a,t-1} \right\}$ and $\left\{ e_{b,t}|b\in M_{a} \right\}$ .

**IF** $M_{a}$ is a singleton comprised of the nearest neighbor $b^{*}$, add $\left( a,t-1 \right)\sim\left( b^{*}, t \right)$ to $L$.

**IF** $\left| M_{a} \right|>1$ **THEN**

Select $b^{*}$ in$M_{a}$ such that the largest number of the edge positions in $e_{a,t-1}$ are associated with those in $e_{b^{*},t}$ in the above.

**ENDIF**

$b^{*}$is added to $A_{t}$.

**ENDIF**

**# One-step ahead prediction:**

Calculate the shifts $d_{i}\in\mathbb{R}^{2} (i=1,\cdots,n)$of the edge positions in $e_{b^{*},t}$, which are assigned to those in $e_{a,t-1}$ ($n$is the number of assigned positions), and the predicted center position is given as

$$\hat{p}_{b^{*},t+1}=p_{b,t}+\left( \alpha/n \right)\sum_{i=1}^{n} d_{i} .$$

**ENDFOR**

ROIs at $t$in which no association is made are added to $A_{t}$, and one-step ahead prediction on the positions are given by the current centers.

**ENDFOR**

**References**

[1] Meyer, F. Topographic distance and watershed lines, *Signal Processing* **38**, 113-125 (1994).

**Optimization of mathematical model.**

We estimate the parameter *γ* and the force function *F* from the experimental data of the time-lapse live imaging of ECs. The interaction between ECs are supposed to be short-range, rather than long-range force. Therefore let us consider the rectangular kernel on bounded support as the density kernel,

$$F\left( \mu\right)=\sum_{k=0}^{N-1} b_{k}\left[ \theta\left( \mu-k\frac{R_{d}}{N} \right)-\theta\left( \mu-\left( k+1 \right)\frac{R_{d}}{N} \right) \right] (S1)$$

$\theta\left( \mu\right)=\left\{ \begin{aligned} 1 \left( \mu\geq0 \right) \\ 0 \left( \mu<0 \right) \end{aligned} \right. \left( S2 \right)$

where $\theta(\mu)$ is the step function, *R_d_* the upper bound distance of cell-cell interaction, and the range [0, *R_d_*] is divided into *N* equal intervals (Figure S5). Through these numerical data, we minimize the following error function *E* for estimation of the parameter *γ* and the function *F* for the isotropic case :

$$E=\sum_{t} \sum_{n} \left\| \left( {\bar{\boldsymbol{v}}}_{n}^{t+1}-{\bar{\boldsymbol{v}}}_{n}^{t} \right)-\left( -\gamma{\bar{\boldsymbol{v}}}_{n}^{t}+\sum_{k\neq n} F\left( \left\| {\bar{\boldsymbol{z}}}_{n}^{t}-{\bar{\boldsymbol{z}}}_{k}^{t} \right\| \right)\frac{{\bar{\boldsymbol{z}}}_{n}^{t}-{\bar{\boldsymbol{z}}}_{k}^{t}}{\left\| {\bar{\boldsymbol{z}}}_{n}^{t}-{\bar{\boldsymbol{z}}}_{k}^{t} \right\|} \right) \right\|^{2} (S3)$$

where ${\bar{\boldsymbol{z}}}_{n}^{t}$ is experimentally obtained cell position of *n*th EC at time *t*, and ${\bar{\boldsymbol{v}}}_{n}^{t}={\bar{\boldsymbol{z}}}_{n}^{t+1}-{\bar{\boldsymbol{z}}}_{n}^{t}$ velocity as numerical difference. Note that the actual time step of the time-lapse was 3 minutes for branch #1 to #4 and 5 minutes for branch #5. Since *E* is the quadratic polynomial of *N* + 1 variables *γ* and $\left\{ b_{i} \right\}_{i=0}^{N-1}$, the minimum of *E* is unique.

This optimization may be considered as multiple linear regression (MLR) since the right hand side of Equation (S3) is linear combination of *γ* and $\left\{ b_{i} \right\}_{i=0}^{N-1}$. In general, given a data set $\left\{ \left( \xi_{i1}, \xi_{i2},\cdots, \xi_{ik};\eta_{i} \right) \right\}_{i=1}^{M}$ and unobserved error variable $\left\{ \varepsilon_{i} \right\}_{i=1}^{M}$, the regression model takes the form $\eta_{i}=\beta_{1}\xi_{i1}+\beta_{2}\xi_{i2}+\cdots+\beta_{k}\xi_{ik}+\varepsilon_{i}$ with regression parameters $\left\{ \beta_{l} \right\}_{l=1}^{k}$. Let the estimation of $\beta_{l}$ be $t_{l}$. Then the minimum of the above error function may be express as $min(E)=\sum_{i=1}^{M} e_{i}^{2}$ where $e_{i}:=\eta_{i}-(t_{1}\xi_{i1}+t_{2}\xi_{i2}+\cdots+t_{k}\xi_{ik})$ is the residual error. Suppose the noise $\varepsilon_{i}$ be i.i.d. normal distribution, and let $X=\left( \xi_{ji} \right)_{ij}$, $\boldsymbol{e}=\left( e_{1},\cdots, e_{M} \right)^{T}$, $\boldsymbol{y}=\left( \eta_{1},\cdots, \eta_{M} \right)^{T}$, $\boldsymbol{t}=\left( t_{1},\cdots, t_{M} \right)^{T}$ where $T$ stands for transposition. Then the MLR model gives the mean $\left\langle\boldsymbol{t} \right\rangle=\left( XX^{T} \right)^{-1}X\boldsymbol{y}$ and the variance $\mathrm{Var}\left( \boldsymbol{t} \right)=\left( XX^{T} \right)^{-1}\left\langle\boldsymbol{e}^{T}\boldsymbol{e} \right\rangle/(M-k)$.

For the anisotropic model, the extended equation based on three cases of two-body interactions on the polarity of EC is described as Equation (S4). The force function *F*_1_ corresponds to two-body force in the case that each velocities of two EC are facing outward with respect to relative positions, *F*_2_ facing inward, and *F*_3_ same direction.

$$v_{x, n}^{t+1}-v_{x, n}^{t}=-\gamma v_{x, n}^{t}+\sum_{k\neq n} F_{1}\left( \left\| \boldsymbol{z}_{n}^{t}-\boldsymbol{z}_{k}^{t} \right\| \right)\frac{x_{n}^{t}-x_{k}^{t}}{\left\| \boldsymbol{z}_{n}^{t}-\boldsymbol{z}_{k}^{t} \right\|} \theta\left( \left( \boldsymbol{z}_{n}^{t}-\boldsymbol{z}_{k}^{t} \right)\cdot\boldsymbol{v}_{n}^{t} \right) \theta\left( -\left( \boldsymbol{z}_{n}^{t}-\boldsymbol{z}_{k}^{t} \right)\cdot\boldsymbol{v}_{k}^{t} \right)+\sum_{k\neq n} F_{2}\left( \left\| \boldsymbol{z}_{n}^{t}-\boldsymbol{z}_{k}^{t} \right\| \right)\frac{x_{n}^{t}-x_{k}^{t}}{\left\| \boldsymbol{z}_{n}^{t}-\boldsymbol{z}_{k}^{t} \right\|} \theta\left( -\left( \boldsymbol{z}_{n}^{t}-\boldsymbol{z}_{k}^{t} \right)\cdot\boldsymbol{v}_{n}^{t} \right) \theta\left( \left( \boldsymbol{z}_{n}^{t}-\boldsymbol{z}_{k}^{t} \right)\cdot\boldsymbol{v}_{k}^{t} \right)+\sum_{k\neq n} F_{3}\left( \left\| \boldsymbol{z}_{n}^{t}-\boldsymbol{z}_{k}^{t} \right\| \right)\frac{x_{n}^{t}-x_{k}^{t}}{\left\| \boldsymbol{z}_{n}^{t}-\boldsymbol{z}_{k}^{t} \right\|} \theta\left( \left( \boldsymbol{z}_{n}^{t}-\boldsymbol{z}_{k}^{t} \right)\cdot\boldsymbol{v}_{n}^{t} \right) \theta\left( \left( \boldsymbol{z}_{n}^{t}-\boldsymbol{z}_{k}^{t} \right)\cdot\boldsymbol{v}_{k}^{t} \right)+\sum_{k\neq n} F_{3}\left( \left\| \boldsymbol{z}_{n}^{t}-\boldsymbol{z}_{k}^{t} \right\| \right)\frac{x_{n}^{t}-x_{k}^{t}}{\left\| \boldsymbol{z}_{n}^{t}-\boldsymbol{z}_{k}^{t} \right\|} \theta\left( -\left( \boldsymbol{z}_{n}^{t}-\boldsymbol{z}_{k}^{t} \right)\cdot\boldsymbol{v}_{n}^{t} \right) \theta\left( -\left( \boldsymbol{z}_{n}^{t}-\boldsymbol{z}_{k}^{t} \right)\cdot\boldsymbol{v}_{k}^{t} \right), (S4)$$

where $\boldsymbol{z}_{n}^{t}=(x_{n}^{t}, y_{n}^{t})$ and $\boldsymbol{v}_{n}^{t}=\boldsymbol{z}_{n}^{t+1}-\boldsymbol{z}_{n}^{t}=(v_{x, n}^{t}, v_{y,n}^{t})$ are cell position and velocity of *n*th EC at time *t*. The relaxation coefficient *γ* and the three function *F*_1_, *F*_2_, and *F*_3_ were estimated by a similar method as above.


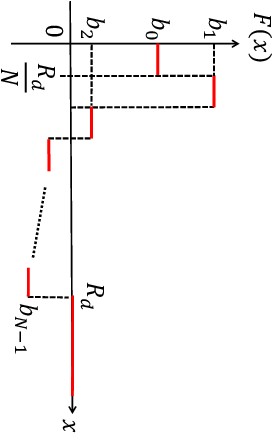


**Figure S5.** Schematic shape of Equation (S1)


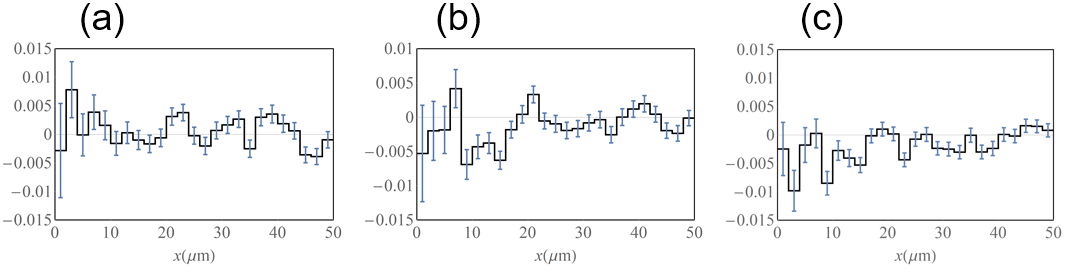


**Figure S6.** Difference of estimated forces (a) $F_{1}\left( x \right)-F_{2}(x)$, (b) $F_{1}\left( x \right)-F_{3}(x)$, (c) $F_{2}\left( x \right)-F_{3}(x)$.


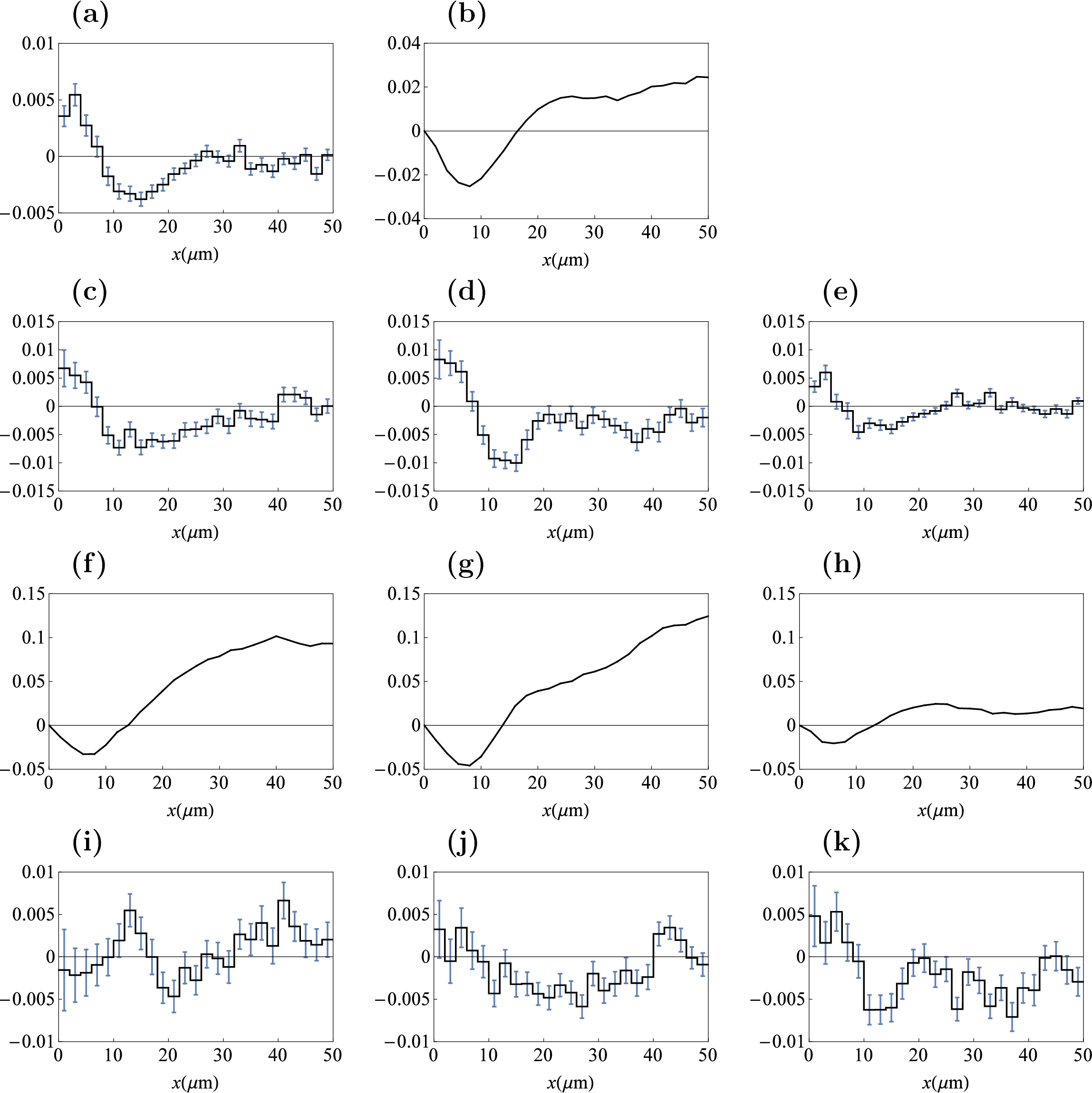


**Figure S7.** [Branch #1] Estimated functions with experimental data of branch #1 (*R_d_* = 50, *N* = 25). For isotropic case, (a) $F\left( x \right)$, (b) $-\int_{0}^{x} F\left( \mu\right)d\mu$. For anisotropic case, (c) $F_{1}\left( x \right)$, (d) $F_{2}\left( x \right)$, (e) $F_{3}\left( x \right)$, and integrals (f) $-\int_{0}^{x} F_{1}\left( \mu\right)d\mu$, (g) $-\int_{0}^{x} F_{2}\left( \mu\right)d\mu$, (h) $-\int_{0}^{x} F_{3}\left( \mu\right)d\mu$, respectively. Difference of estimated forces (i) $F_{1}\left( x \right)-F_{2}\left( x \right)$, (j) $F_{1}\left( x \right)-F_{3}\left( x \right)$, (k) $F_{2}\left( x \right)-F_{3}\left( x \right)$, respectively.


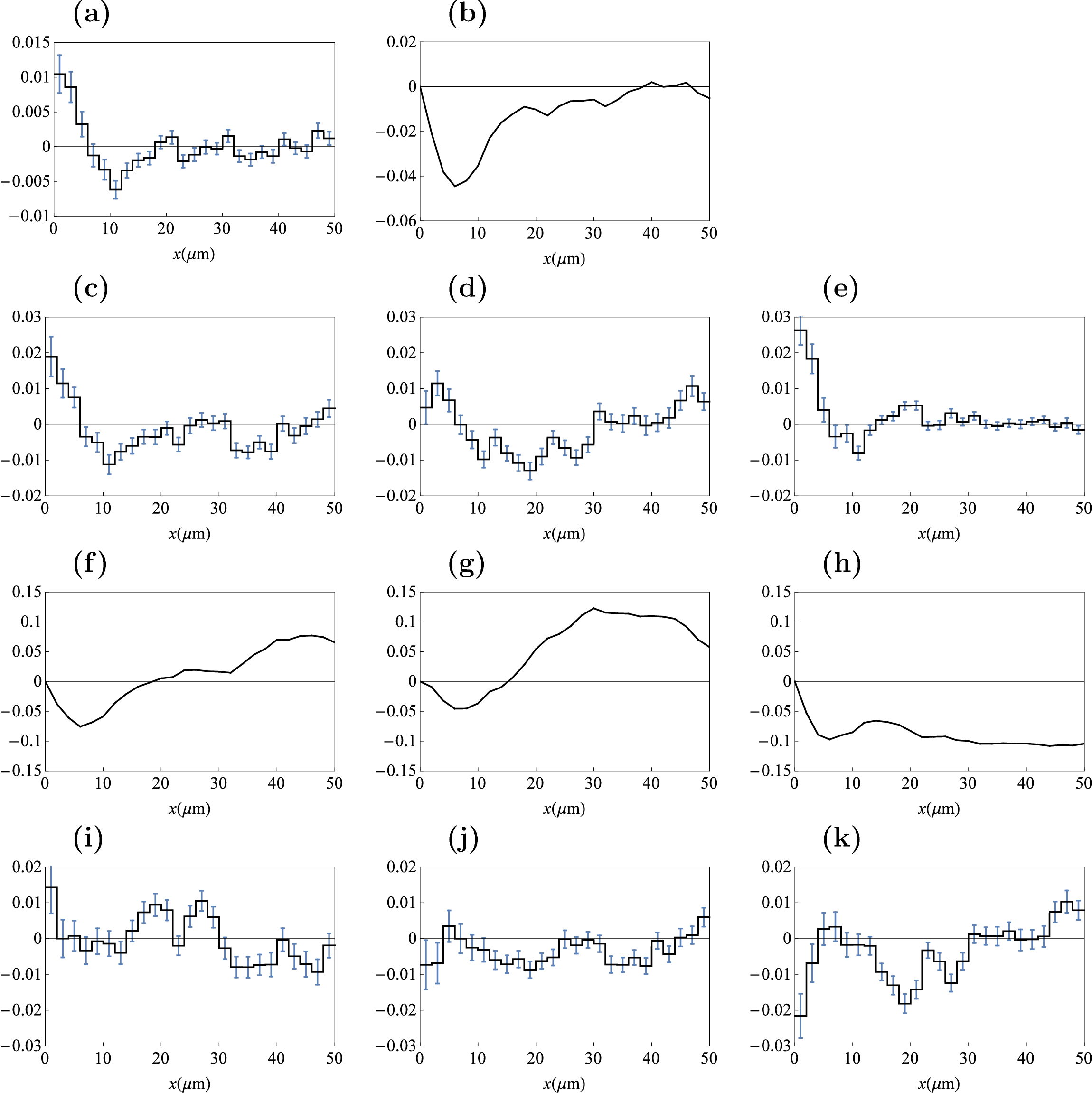


**Figure S8.** [Branch #3] Estimated functions with experimental data of branch #3 (*R_d_* = 50, *N* = 25). For isotropic case, (a) $F\left( x \right)$, (b) $-\int_{0}^{x} F\left( \mu\right)d\mu$. For anisotropic case, (c) $F_{1}\left( x \right)$, (d) $F_{2}\left( x \right)$, (e) $F_{3}\left( x \right)$, and integrals (f) $-\int_{0}^{x} F_{1}\left( \mu\right)d\mu$, (g) $-\int_{0}^{x} F_{2}\left( \mu\right)d\mu$, (h) $-\int_{0}^{x} F_{3}\left( \mu\right)d\mu$, respectively. Difference of estimated forces (i) $F_{1}\left( x \right)-F_{2}\left( x \right)$, (j) $F_{1}\left( x \right)-F_{3}\left( x \right)$, (k) $F_{2}\left( x \right)-F_{3}\left( x \right)$, respectively.


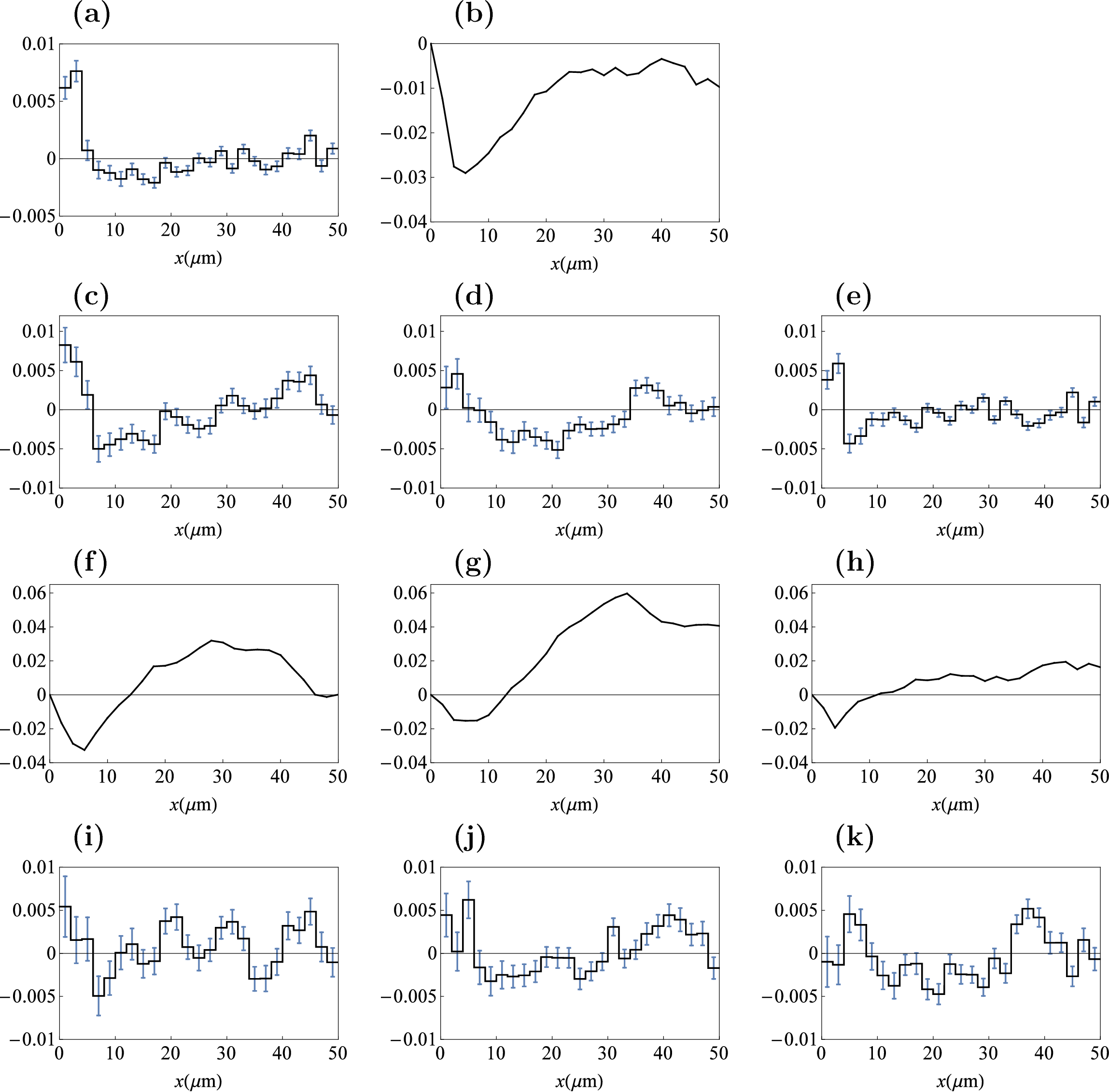


**Figure S9.** [Branch #4] Estimated functions with experimental data of branch #4 (*R_d_* = 50, *N* = 25). For isotropic case, (a) $F\left( x \right)$, (b) $-\int_{0}^{x} F\left( \mu\right)d\mu$. For anisotropic case, (c) $F_{1}\left( x \right)$, (d) $F_{2}\left( x \right)$, (e) $F_{3}\left( x \right)$, and integrals (f) $-\int_{0}^{x} F_{1}\left( \mu\right)d\mu$, (g) $-\int_{0}^{x} F_{2}\left( \mu\right)d\mu$, (h) $-\int_{0}^{x} F_{3}\left( \mu\right)d\mu$, respectively. Difference of estimated forces (i) $F_{1}\left( x \right)-F_{2}\left( x \right)$, (j) $F_{1}\left( x \right)-F_{3}\left( x \right)$, (k) $F_{2}\left( x \right)-F_{3}\left( x \right)$, respectively.


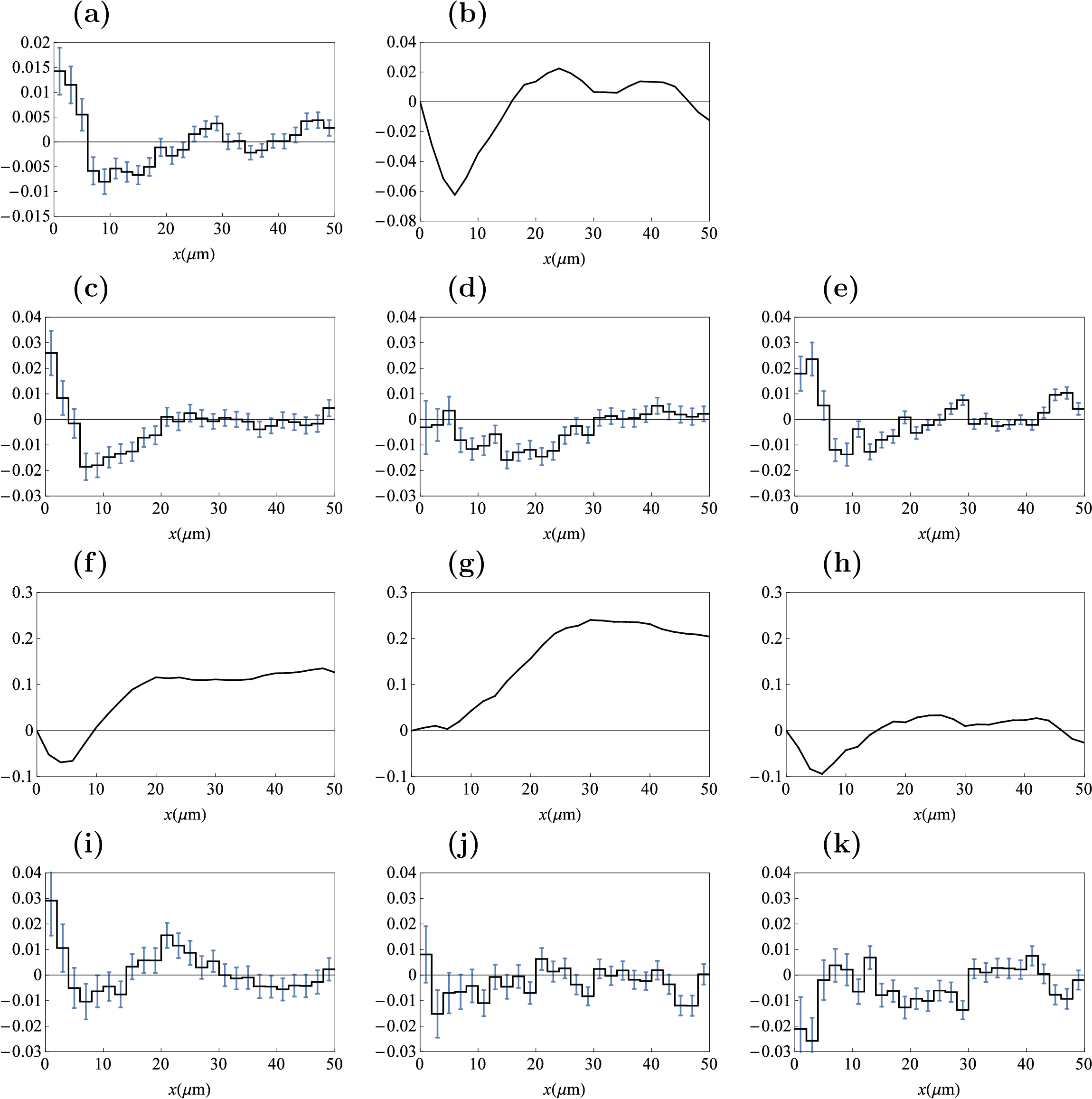


**Figure S10.** [Branch #5] Estimated functions with experimental data of branch #5 (*R_d_* = 50, *N* = 25). For isotropic case, (a) $F\left( x \right)$, (b) $-\int_{0}^{x} F\left( \mu\right)d\mu$. For anisotropic case, (c) $F_{1}\left( x \right)$, (d) $F_{2}\left( x \right)$, (e) $F_{3}\left( x \right)$, and integrals (f) $-\int_{0}^{x} F_{1}\left( \mu\right)d\mu$, (g) $-\int_{0}^{x} F_{2}\left( \mu\right)d\mu$, (h) $-\int_{0}^{x} F_{3}\left( \mu\right)d\mu$, respectively. Difference of estimated forces (i) $F_{1}\left( x \right)-F_{2}\left( x \right)$, (j) $F_{1}\left( x \right)-F_{3}\left( x \right)$, (k) $F_{2}\left( x \right)-F_{3}\left( x \right)$, respectively.


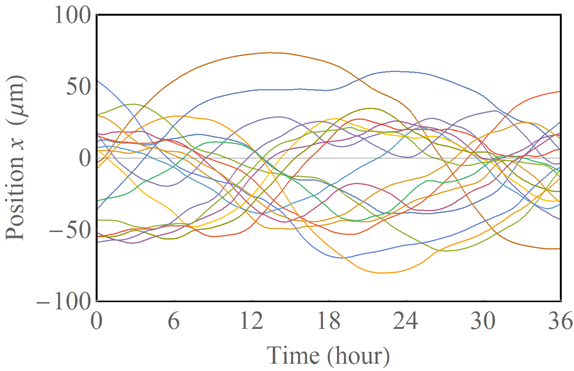


**Figure S11.** Simulational results of time evolutions of individual EC positions along each branch elongation (*x*-axis). The parameter was optimized in accordance with experimental results of branch #2 (Figure 6). Each line with different color represents trajectory of individual EC.

**Movie S1.** Fluorescence movie tracking of individual EC nuclei in an elongating branch using the tracking system.

**Movie S2.** Fluorescence movie tracking of nucleus of a U-turn cell in an elongating branch using the tracking system.

**Movie S3(a)-(c).** Fluorescence movies 4-6 hours after excision of the aortic sheet of individual EC nuclei in different parts of isolated branches. Yellow line shows original boundary between an aortic sheet and new branches.

**Movie S4(a)-(e).** Fluorescence movies of individual EC nuclei in different parts of isolated branches.
